# Glucose deprivation increases tau phosphorylation via P38 mitogen‐activated protein kinase

**DOI:** 10.1111/acel.12381

**Published:** 2015-07-29

**Authors:** Elisabetta Lauretti, Domenico Praticò

**Affiliations:** ^1^ Department of Pharmacology and Center for Translational MedicineTemple University School of MedicinePhiladelphiaPA19140USA

**Keywords:** Alzheimer's disease, amyloid beta, glucose deprivation, mitogen‐activated protein kinase, neuronal cells, tau phosphorylation

## Abstract

Alterations of glucose metabolism have been observed in Alzheimer's disease (AD) brain. Previous studies showed that glucose deprivation increases amyloidogenesis via a BACE‐1‐dependent mechanism. However, no data are available on the effect that this condition may have on tau phosphorylation. In this study, we exposed neuronal cells to a glucose‐free medium and investigated the effect on tau phosphorylation. Compared with controls, cells incubated in the absence of glucose had a significant increase in tau phosphorylation at epitopes Ser202/Thr205 and Ser404, which was associated with a selective activation of the P38 mitogen‐activated protein kinase. Pharmacological inhibition of this kinase prevented the increase in tau phosphorylation, while fluorescence studies revealed its co‐localization with phosphorylated tau. The activation of P38 was secondary to the action of the apoptosis signal‐regulating kinase 1, as its down‐regulation prevented it. Finally, glucose deprivation induced cell apoptosis, which was associated with a significant increase in both caspase 3 and caspase 12 active forms. Taken together, our studies reveal a new mechanism whereby glucose deprivation can modulate AD pathogenesis by influencing tau phosphorylation and suggest that this pathway may be a new therapeutic target for AD.

**Abbreviations**
AD: Alzheimer's disease
NFT: neurofibrillary tangles
CNS: central nervous system
Aβ: amyloid beta
APP: amyloid beta precursor protein
BACE‐1: beta secretase‐1
GSK3: glycogen synthase kinase 3
CDK5: cyclin kinase 5
MAPK: mitogen‐activated protein kinase
ASK1: apoptosis signal‐regulating kinase 1
DMEM: Dulbecco's modified Eagle's medium

## Introduction

Alzheimer's disease (AD) is an age‐related neurodegenerative disorder with dementia characterized by progressive accumulation of amyloid beta (Aβ) peptides and abnormal aggregates of the microtubule‐associated tau protein, also known as neurofibrillary tangles (NFTs) (Giannopoulos & Praticò, 2015). Recent functional evidence suggests that Aβ initiates the AD changes, but pathological tau protein causes neurodegeneration and better correlates with the clinical manifestation of the disease (Arriagada *et al*., 1992; Riley *et al*., 2002). While we know that NFTs are mainly composed of abnormally hyperphosphorylated tau that aggregates into paired helical filaments and straight filaments, the mechanisms leading to the abnormal hyperphosphorylation of tau in the brain of AD patients remain unclear.

Glucose is the main source of energy in the central nervous system (CNS), as under physiological conditions neuronal cells are entirely dependent on a continuous supply of glucose for their correct functioning. The CNS is particularly vulnerable to hypoglycemic damage, and changes in glucose metabolism have been observed in a variety of neurodegenerative conditions associated with dementia (Peppard *et al*., 1990, 1992; De Leon *et al*., 2007; Mosconi *et al*., 2007). In particular, positron emission tomography imaging studies have shown that glucose utilization is dramatically lower in AD, compared to aged‐matched, nondemented brain (Mosconi *et al*., 2009; Landau *et al*., 2012). Moreover, postmortem analysis of AD brain shows down‐regulated expression of mitochondrial enzymes indicating a deficiency in energy metabolism (Soane *et al*., 2007).

In line with this concept, previous studies have demonstrated that using a pharmacological model of energy metabolism inhibition in APP overexpressing transgenic mice (i.e., Tg2576), BACE‐1 and Aβ levels become elevated, suggesting that energy deprivation may be amyloidogenic *in vivo* (O'Connor *et al*., 2008).

However, to the best of our knowledge no data are available on the effect that a dysregulation of glucose levels may have on tau and its phosphorylation state. To address this important biological question in the current study, we exposed neuronal cells to a glucose‐free medium and investigated the effect on tau phosphorylation and the potential mechanisms involved in this biological effect. Under this experimental condition, we found that, compared with controls, glucose‐deprived cells had significant increase in tau phosphorylation at specific epitopes, which was dependent on the activation of the P38 mitogen‐activated protein kinase (MAPK) pathway.

## Results

### Glucose deprivation induces tau phosphorylation

**Figure 1:**
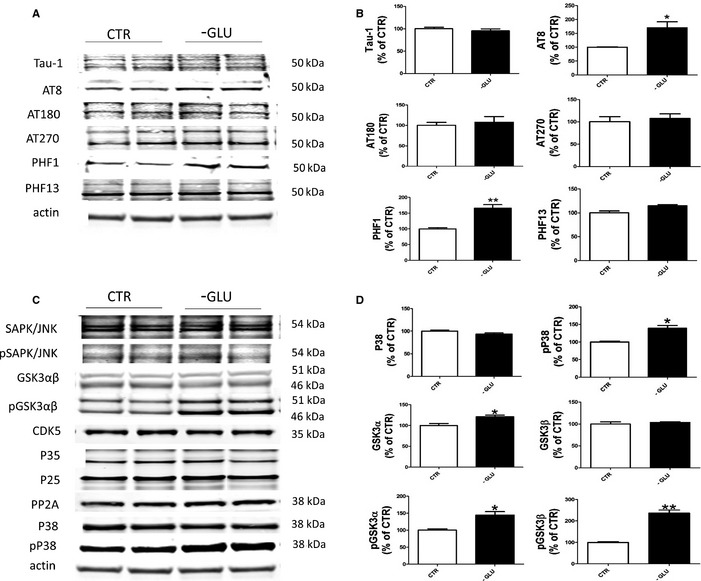
Glucose deprivation modulates tau phosphorylation in neuronal cells. (A) Representative Western blot analyses for total tau (Tau‐1) and phosphorylated tau at residues S202/T205 (AT8), T231/S235 (AT180), T181 (AT270), S396/S404 (PHF‐1), and S396 (PHF‐13) in lysates from neuronal cells incubated 24 h in either normal DMEM (CTR) or DMEM without glucose (‐GLU). (B) Densitometric analyses of the immunoreactivities shown in the previous panel (*n* = 6; *P < 0.05; **P < 0.001). (C) Representative Western blot analyses for (SAPK)/JNK and (pSAPK)/JNK, GSK‐3α, GSK‐3β, pGSK‐3α, pGSK3β, CDK5, P35 P25, PP‐2A, P38, and pP38 in lysates from neuronal cells incubated 24 h in either normal DMEM (CTR) or DMEM without glucose (‐GLU). (D) Densitometric analyses of the immunoreactivities shown in the previous panel (*n* = 6; *P < 0.05; **P < 0.001). Results are mean ± SEM.

N2A cells were incubated for 24 h in either DMEM with glucose, or DMEM without glucose. Compared with controls, no changes in levels of total tau were observed in cells that were exposed to medium without glucose (Fig. 1A). However, glucose‐deprived cells had a significant increase in tau phosphorylation at Ser202/Thr205 as recognized by the antibody AT8, and at Ser404 as recognized by the antibody PHF1. By contrast, compared with controls, the absence of glucose did not alter the phosphorylation status at Thr231/Ser235 as recognized by the antibody AT180, at Thr‐181 as recognized by the antibody AT270, and at Ser396 as recognized by the antibody PHF13 (Fig. 1A).

To study the mechanism underlying the increase in tau phosphorylation after glucose deprivation, we next examined some of the kinases and phosphatases which are considered major regulators of this post‐translational modification. As shown in Figure 1C, we did not find any changes in the levels of cyclin kinase‐5 (CDK5) and its two coactivators P35 and P25, phosphorylated stress‐activated protein kinase (SAPK)/JNK and the phosphatase 2A (PP2A). By contrast, compared with controls, while no change was observed for the total level of P38 MAPK, glucose‐deprived cells shown a significant increase in its active form (phosphorylated at Thr180 and Tyr182) (pP38). Moreover, the absence of glucose elevated the total level of glycogen synthase kinase 3‐α (GSK3‐α) and phosphorylated (p) GSK3‐β and pGSK3‐α (Fig. 1C, D).

### P38 mitogen‐activated protein kinase mediates glucose deprivation‐induced tau phosphorylation

**Figure 2:**
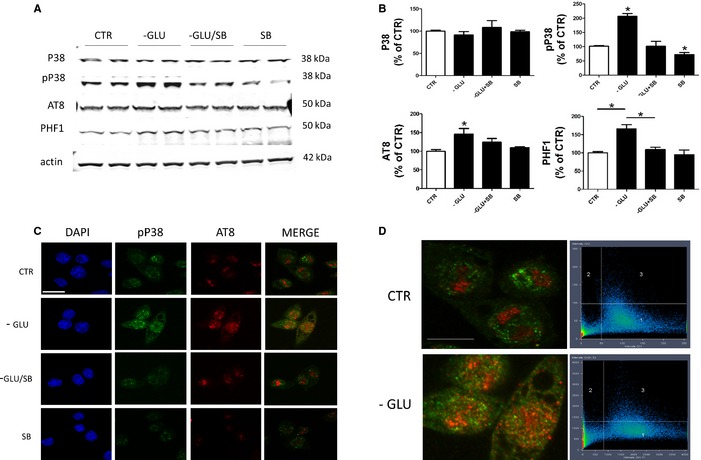
P38 mitogen‐activated protein kinase mediates glucose deprivation‐induced tau phosphorylation. (A) Representative Western blot analyses of P38, pP38, phosphorylated tau at residues S202/T205 (AT8), and S396/S404 (PHF‐1), in lysates from cells treated with vehicle or SB20358 (20 μm) (SB), in either normal DMEM (CTR) or DMEM without glucose (‐GLU). (B) Densitometric analyses of the immunoreactivities to the antibodies presented in the previous panel (*n* = 4; *P = 0.05). Results are mean ± SEM. (C) Representative images of immunofluorescence analysis for pP38 and AT8 in neuronal cells treated with vehicle or SB20358 (20 μm) (SB), in either normal DMEM (CTR) or DMEM without glucose (‐GLU) (scale bar: 20 μm). (D) Quantitative analysis of the immune co‐localization signal for pP38 and AT8 as observed in panel C.

To provide evidence for a functional role of P38 activation in the glucose deprivation‐induced tau phosphorylation, cells were incubated with SB20358, a specific inhibitor of this kinase activation (20 μm) (Katome *et al*., 2013). As shown in Figure 2A, we found that the pharmacological blockade of P38 activation prevented the energy deprivation‐dependent hyperphosphorylation of tau. These results were confirmed by performing double staining immunofluorescence assays. The pP38 was detected by green fluorescent signal and phosphorylated tau as the red fluorescent signal under a fluorescent microscope. As shown in Figure 2C, while glucose‐deprived cells had an increase in the signal for the antibody AT8, which recognizes tau phosphorylated at Ser202/Thr205, this effect was blunted when cells were incubated with the specific P38 inhibitor. The merged images (Fig. 2D) showed that the green fluorescent for pP38 co‐localized with the red fluorescent for AT8 antibody.

**Figure 3:**
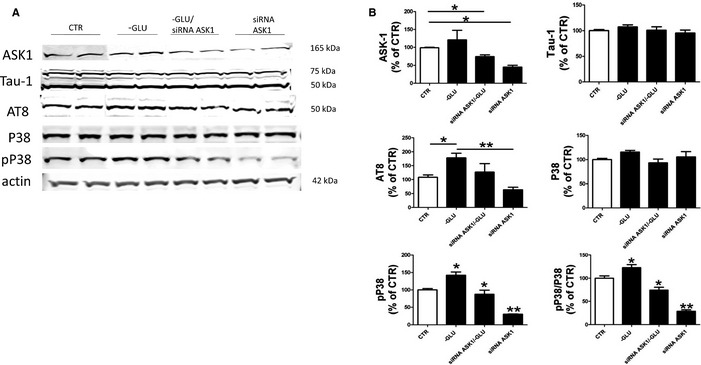
Involvement of ASK1 in the glucose deprivation‐induced P38 activation and tau phosphorylation. (A) Representative Western blot analysis for ASK1, total tau (Tau‐1), and tau phosphorylated at S202/T205 as recognized by the antibodies AT8, P38, and pP38 in lysates from neuronal cells preincubated with siRNA ASK1 (0.1 μmol L^‐1^) and then challenged with glucose‐free media (‐GLU) or regular medium (CTR). (B) Densitometric analyses of the immunoreactivities shown in the previous panel (*n* = 4; *P < 0.05; **P < 0.005). Results are mean ± SEM.

As it is known that stress‐dependent P38 activation requires the participation of the apoptosis signal‐regulating kinase 1 (ASK1), next we investigated the involvement of this kinase under our experimental conditions. To this end, we down‐regulated ASK1 protein levels using a siRNA approach and found that the reduced availability of this kinase in glucose‐deprived cells was sufficient to prevent the phosphorylation/activation of P38 MAPK (Fig. 3). Under the same experimental conditions, we also observed that while total tau levels were unchanged, glucose deprivation‐dependent tau phosphorylation as recognized by the AT8 antibody was prevented (Fig. 3).

### P38 mitogen‐activated protein kinase inhibition rescues glucose deprivation‐induced apoptosis

**Figure 4:**
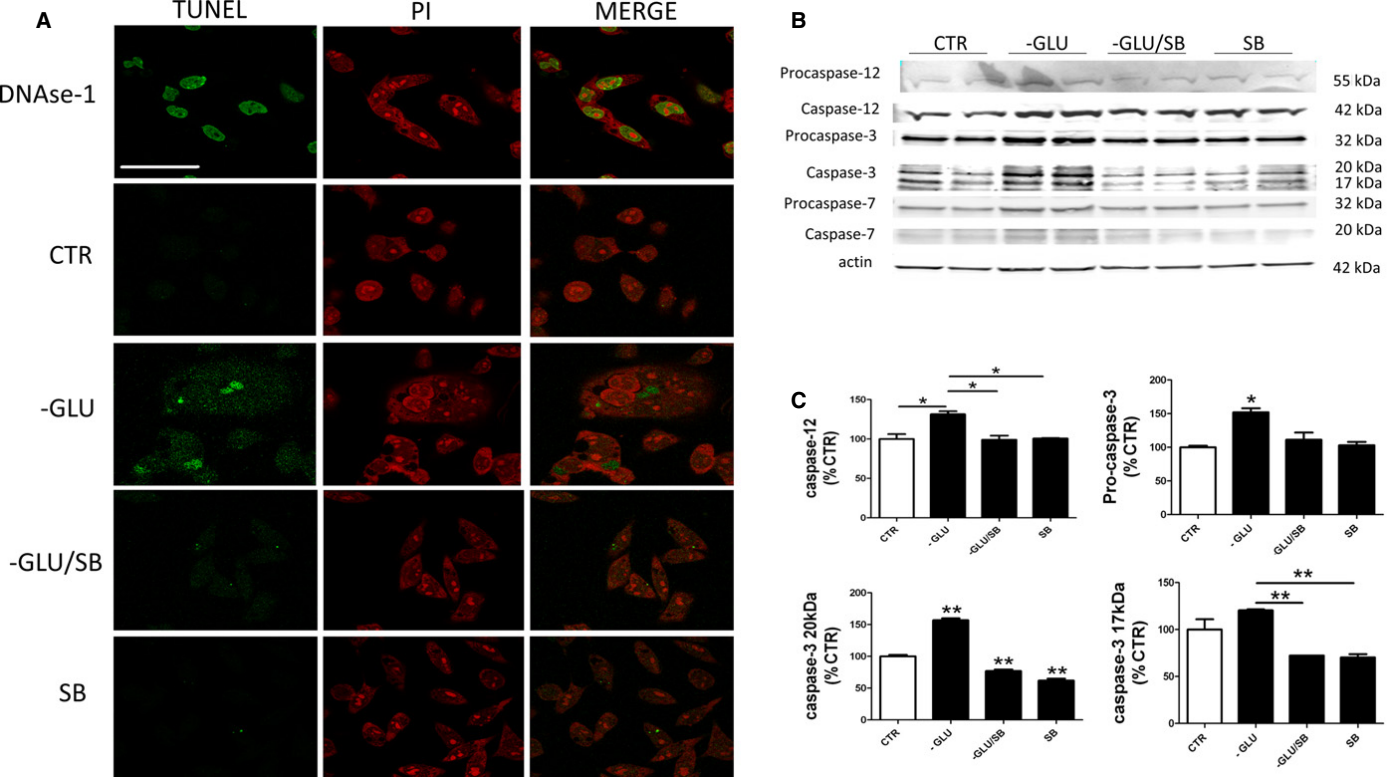
Glucose deprivation‐dependent neuronal apoptosis is mediated by caspases 12 and 3. (A) TUNEL assay. N2A cells treated with vehicle or SB20358 (20 μm) (SB), in either normal DMEM (CTR) or DMEM without glucose (‐GLU), were subjected to TUNEL assay and counterstained with propidium iodide (PI). Positive controls were prepared using DNase I (scale bar: 50 lm). (B) Representative Western blot analysis for procaspase‐12, procaspase‐3, procaspase‐7, caspase‐12, caspase‐3, and caspase‐7 in lysates from neuronal cells treated with vehicle or SB20358 (20 μm) (SB) in normal DMEM (CTR) or glucose‐free DMEM (‐GLU). (C) Densitometric analyses of the immunoreactivities are shown in the previous panel (*n* = 4; *P < 0.05; **P < 0.001). Results are mean ± SEM.

As stress activation of the P38 MAPK pathway can also lead to cell apoptosis, next we assayed our cells for immune‐cytochemical evidence of it. As shown in Figure 4A, immunofluorescence studies revealed that compared with controls, glucose‐deprived cells had an increase in apoptosis as indicated by the increase in TUNEL staining. By contrast, this staining was not detectable in the presence of SB20358, the specific P38 inhibitor. In search for the mediators of the apoptotic response, we investigated some of the caspases which have been involved in this biological effect (Nakagawa *et al*., 2000; Morishima *et al*., 2002). Compared with controls, glucose‐deprived cells showed a significant increase in the level of caspase‐3 and caspase‐12, which were blunted in the presence of the P38 inhibitor (Fig. 4B,C). By contrast, no significant differences were observed between the two conditions when procaspase‐12, pro‐caspase‐7, and caspase 7 steady‐state levels were assayed (Fig. 4B,C).

## Discussion

Numerous clinical studies have shown that there is a biological link between impaired energy metabolism, glucose utilization, and AD pathogenesis (Craft & Watson, 2004; Steen *et al*., 2005), which has been confirmed in both cellular and animal models of energy deprivation. Thus, using a pharmacological model of energy metabolism inhibition in Tg2576 mice, Velliquette *et al*. reported that Aβ levels were significantly increased suggesting for the first time that energy deprivation acts as an amyloidogenic stimulus *in vivo* (Velliquette *et al*., 2005). In a later paper that used glucose deprivation in cell culture as a model of energy deficiency, it was demonstrated a post‐transcriptional increase in BACE1 level and enhanced Aβ production (O'Connor *et al*., 2008). This effect was then confirmed in two APP transgenic mouse models (i.e., Tg2576 and 5xFAD mice) in which pharmacological energy deprivation promoted amyloidogenesis via a BACE‐1‐dependent mechanism (O'Connor *et al*., 2008).

However, despite this evidence no data are available on the effect that impaired energy metabolism may have on tau phosphorylation. In the current paper, we provide the first *in vitro* experimental proof that in response to the inhibition of energy metabolism, recreated by a condition of glucose deprivation, neuronal cells manifest a significant increase in tau phosphorylation, which is mediated by the activation of the P38 MAP kinase.

Taken together, our data lend further support to a growing amount of literature showing the central role that different types of cellular stress responses could play in the onset and development of AD neuropathology. The discovery of an involvement of the P38 MAP kinase in this biological effect does not come as surprise considering that this very kinase activation has been observed in response to a variety of extracellular stimuli and stressors such as UV light, heat, osmotic shock, inflammatory cytokines, and growth factors (Munoz & Ammit, 2010). Of interest, it is the other observation that the active phosphorylated form of this kinase has been found to be physically associated with hyperphosphorylated tau within the NFT of AD brain (Munoz & Ammit, 2010).

Although the normal roles of Aβ and tau in the CNS are not completely understood, one of the several proposed possibilities is that both may be involved in response to stress or external stimuli (Tesco *et al*., 2007; Wen *et al*., 2008). Impaired energy metabolism is definitely one type of stress that is responsible of modifying APP processing and elevating Aβ formation via a translational control of BACE1. However, whether energy deprivation is capable to alter tau metabolism and its post‐translational modification is still not demonstrated.

With this goal in mind, in our studies we use a well‐established model of impaired energy metabolism by incubating neuronal cells in a medium not containing glucose. At the end of this treatment, we observed a significant increase in tau phosphorylation at the epitopes recognized by the AT8 and PHF1 antibodies, but not at any of the other epitopes investigated and recognized by AT180, AT270, and PHF13 antibodies, suggesting the specific nature of this biological effect. In search for the molecular mechanism that could be responsible for this selective increase in tau phosphorylation, we assayed several of the kinases that are considered important modulators of tau post‐translational modification. Among them, we observed that pGSK3α and pGSK3β were elevated in cells incubated in glucose‐free medium. We interpret these changes in the activation state of this kinase isoforms as not relevant to the increase in tau phosphorylation we observed under our experimental conditions as it is known that an increase in pGSK3 is an index of a reduced activity of this kinase (Fang *et al*., 2000). For this reason, we believe that, as previously reported, the change in pGSK3 in our cells is a generic response to an energy‐deprived condition (Planel *et al*., 2001).

In recent years, the P38 MAP kinase has attracted much attention as an important regulator of tau phosphorylation. P38 exists in four isoforms (α, β, γ, δ), all implicated in tau phosphorylation when activated by dual phosphorylation at Thr180 and Tyr182. Several studies have shown that P38 active form (i.e., pP38) is associated with neuritic Aβ‐amyloid plaques and tau NFT in postmortem brains of AD patients and that P38 activation occurs at very early stage of the disease (Reynolds *et al*., 2000; Peel *et al*., 2004).

Interesting in our studies we documented not only an activation of this kinase but importantly its co‐localization with a phosphorylated tau isoform which is an early component of full‐blown tau pathology, that is, NFTs. In addition, using a selective and specific pharmacological inhibitor, we were able to prove that the activation of this kinase played a functional role in the glucose deprivation‐dependent changes in tau phosphorylation.

As stress can activate ASK1, a protein kinase of the MAPK kinase family that in turn activates the P38 MAPK signaling cascade, we were interested to investigate whether this was the case in our cells (Tobiume *et al*., 2001). Thus, by down‐regulating ASK1 mRNA and significantly reducing its protein levels, we were able to show that even in the presence of a glucose‐free medium, neuronal cells did not show changes in pP38 and most importantly, the intracellular levels of tau phosphorylated were also unmodified compared with controls.

Accumulating evidence suggests that ASK1 may contribute to the pathogenesis of AD and other neurodegenerative disorders by regulating various cellular responses such as apoptosis, cell survival, and differentiation (Song *et al*., 2014).

While ASK1 is known to be activated by several internal or external environmental stressors, the extent and duration of the exposure to them is crucial to determine subsequent cell fate. In fact, ASK1 activation as result of short or low stress exposure leads to cell survival/differentiation; by contrast, prolonged exposure leads to apoptosis (Matsuzawa *et al*., 2002).

In our studies, because of the prolonged duration (24‐h glucose deprivation) of the *in vitro* stress exposure, the activation of ASK1 was associated with cell apoptosis, which was accompanied by the activation of caspase‐3 and caspase‐12. Importantly, this activation was prevented by the pharmacological inhibition of P38 kinase activity, further supporting the role of this pathway in the cell apoptotic response to the impaired energy metabolism secondary to glucose unavailability.

## Conclusions

In summary, our work using a model of energy deficiency provides the first *in vitro* experimental evidence that in response to a condition of glucose deprivation stress, neuronal cells manifest an increase in tau phosphorylation and apoptotic response which were mediated by the activation of the P38 MAPK pathway via the participation of the ASK1 kinase.

Considering that impaired glucose utilization is a well‐known AD risk factor, the targeting of this kinase could afford a new therapeutic opportunity for developing preventative and disease‐modifying therapies for AD.

## Experimental procedures

### Cell culture

The N2A (neuro‐2 A neuroblastoma) neuronal cells stably expressing human APP carrying the K670 N, M671 L Swedish mutation (APPswe), were grown in Dulbecco's modified Eagle medium (DMEM) (cat. # 11965‐092; Gibco, Grand Island, NY, USA) supplemented with 10% fetal bovine serum, 100 U mL^−1^ penicillin, 100 μg mL^−1^ streptomycin, and 400 μg mL^−1^ G418 (Gibco), at 37 °C in the presence of 5% CO_2_, as previously described (Lauretti *et al*., 2015). For each experiment, equal numbers of cells were plated in six‐well plates; the day of the experiment media was removed, cells washed with PBS and fresh glucose‐free media (cat. #11966‐025, Gibco) or regular media were (cat. #11965‐092, Gibco) added. After 24‐h incubation, cell pellets were harvested in lytic buffer for immunoblot analyses as described below.

### Western blot analysis

Proteins were extracted in EIA buffer containing 250 mm Tris base, 750 mm NaCl, 5% NP‐40, 25 mm EDTA, 2.5% sodium deoxycholate, 0.5% SDS, and an EDTA‐free protease and phosphatase inhibitors cocktail tablet (Roche Applied Science, Indianapolis, IN, USA). Samples were centrifuged at 15.7 g for 20 min at 4 °C, and supernatants were used for immunoblot analysis, as previously described (Di Meco *et al*., 2014). Total protein concentration was determined using BCA Protein Assay Kit (Pierce, Rockford, IL, USA). Samples were electrophoretically separated using 10% Bis‐Tris gels (Bio‐Rad, Richmond, CA, USA), according to the molecular weight of the target molecule, and then transferred onto nitrocellulose membranes (Bio‐Rad). They were blocked with Odyssey blocking buffer for 1 h and then incubated with the appropriate primary antibodies as described in the Table 1 overnight at 4 °C. After three washing cycles with T‐TBS, membranes were incubated with IRDye 800CW or IRDye 680CW‐labeled secondary antibodies (LI‐COR Bioscience, Lincoln, NE, USA) at 22 °C for 1 h. Signals were developed with Odyssey Infrared Imaging Systems (LI‐COR Bioscience). Actin was always used as an internal loading control.

**Table 1:** Antibodies used in the study

Antibody	Immunogen	Cat #	Host	Application	Source
Tau‐1	Bovine microtubule‐associated protein	4019	Mouse	WB	Cell Signaling
AT8	Peptide containing phospho‐S202/T205	MN1020	Mouse	WB, IF	Thermo Scientific
AT180	Peptide containing phospho‐T231/S235	MN1040	Mouse	WB,	Thermo Scientific
AT270	Peptide containing phospho‐T181	MN1050	Mouse	WB,	Thermo Scientific
PHF13	Peptide containing phospho‐Ser396	9632	Mouse	WB	Cell Signaling
PHF1	Peptide containing phospho‐Ser396/S404		Mouse	WB	Dr. P. Davies
GSK3α/β	aa 1‐420 full‐length GSK3β of Xenopus origin	sc‐9166	Mouse	WB	Santa Cruz
pGSK3αβ	aa around Ser21 of human GSK3α	9331	Rabbit	WB	Cell Signaling
SAPK/JNK	aa of human NK2	9252	Mouse	WB	Cell Signaling
pSAPK/JNK	aa of recombinant human JNK2 fusion protein	9255	Rabbit	WB	Cell Signaling
CDK5	aa C‐terminus of CDK5 of human origin	sc‐173	Rabbit	WB	Santa Cruz
P35/P25	aa C‐terminus of P35/25 of human origin	Sc‐820	Rabbit	WB	Santa Cruz
PP2A	aa 295‐309 of catalytic subunit of human protein phosphatase 2A. Clone 1D6	PA5‐17510	Mouse	WB	Thermo Scientific
P38	Peptide corresponding to the sequence of human P38	9212	Rabbit	WB	Cell Signaling
Pp38	Phospho‐peptide corresponding to residues surrounding Thr180/Tyr182 of human P38	4511	Rabbit	WB, IF	Cell Signaling
Caspase‐12	Aa around 158 of mouse caspase‐12	2202	Rabbit	WB	Cell Signaling
Caspase‐3	Aa 1‐277 of full‐length procaspase‐3 of human origin	sc‐7148	Rabbit	WB	Santa Cruz
Caspase‐7	Aa 15‐50 near the N‐term of caspase‐7 of human origin	sc‐28295	Mouse	WB	Santa Cruz
ASK1	Aa 1076‐1375 of ASK1 of human origin	sc‐7931	Rabbit	WB	Santa Cruz

WB, Western blot; IF, immunofluorescence.

### Cell treatments

To test the effect of a P38 MAPK inhibitor on tau phosphorylation, N2A‐APPswe cells were grown to 70% confluence, then incubated for 1 h with the P38 MAPK inhibitor (SB 203580; Millipore, Billerica, MA, USA) (20 μm) prior incubation with the glucose‐free medium (DMEM) and then treated with the same inhibitor for additional 24 h, after which cells pellets were harvested in lytic buffer for biochemical analyses. In another set of experiment, before the incubation with the glucose‐deprived medium, cells were treated overnight with small interfering (si) RNA for ASK1 (Ambion, Life Technology, Grand Island, NY, USA).

### Immunofluorescence microscopy

Cell immunostaining was carried out as previously described (Chu *et al*., 2015). Briefly, after treatment, N2A‐APPswe cells were plated on glass coverslips, and the following day, fixed in 4% paraformaldehyde in PBS for 15 min at 22 °C. After rinsing several times with PBS, cells were incubated in a blocking solution (5% normal serum/0.4% TX‐100) for 1 h at 22 °C and then with the primary antibody against AT8 (1:50; Thermo Scientific, Rockford, IL, USA), and the active form of P38 kinase (1:100 Cell Signaling, Danvers, MA, USA) overnight at 4 °C. After several washings with PBS, cells were incubated for 1 h with a secondary Alexa 488‐conjugated antibody (1:200; Invitrogen, Camarillo, CA, USA). Coverslips were mounted using VECTASHIELD mounting medium (Vector Laboratories, Burlingame, CA, USA) and analyzed with a confocal microscope (Zeiss 710, Carl Zeiss Microscopy GmbH, Carl Zeiss Promenade, Jena, Germany) with a 639 oil objective and sequential scanning at 488 and 561 nm. Control coverslips were processed as described above except that no primary antibody was added to the solution.

Tunel assay was carried out following the manufacturer's instructions (DeadEndTM Fluorimetric TUNEL system; Promega, Madison, WI, USA). Briefly, cells were fixed with a cross‐linking fixative such as 4% methanol‐free formaldehyde and then permeabilized by suspending them in 0.2% Triton X‐100 solution in PBS for 5 min. Cells were then incubated with the terminal deoxynucleotidyl transferase recombinant enzyme (rTdT) for 60 min at 37 °C. The 3′ OH‐termini of the DSBs serve as primers and become labeled in this procedure with fluorescin‐12‐dUTP when incubated in the reaction catalyzed by rTdT. Positive controls were prepared using DNase I. Concurrent staining of DNA with propidium iodide was applied. Coverslips were mounted using VECTASHIELD mounting medium (Vector Laboratories) and analyzed with the confocal microscope described above.

### Data analysis

One‐way anova followed by Bonferroni's multiple comparison tests were performed using graphpad prism 5.0 (GraphPad Software, La Jolla, CA, USA). All data are presented as mean ± S.E.M. Significance was set at *P* < 0.05.

## Authors' contributions

E.L. and P.D. designed the study. E.L. executed all the experiments. E.L. and D.P. interpreted the data and wrote the manuscript. Both authors read and approved the final version of the manuscript.

## Funding

No funding information provided.

## Conflict of interest

The authors declare no competing interest to disclose.
